# Serum Zinc and Selenium Concentrations in Patients with Hypertrophy and Remodelling of the Left Ventricle Secondary to Arterial Hypertension

**DOI:** 10.3390/antiox10111803

**Published:** 2021-11-12

**Authors:** Paweł Gać, Karolina Czerwińska, Małgorzata Poręba, Adam Prokopowicz, Helena Martynowicz, Grzegorz Mazur, Rafał Poręba

**Affiliations:** 1 Department of Population Health, Division of Environmental Health and Occupational Medicine, Wroclaw Medical University, 50-368 Wroclaw, Poland; karolina.czerwinska@student.umw.edu.pl; 2 Department of Paralympic Sports, Wroclaw University of Health and Sport Sciences, 51-617 Wroclaw, Poland; poreba1@wp.pl; 3 Institute of Occupational Medicine and Environmental Health in Sosnowiec, 41-200 Sosnowiec, Poland; adam.prokopowicz@interia.com; 4 Department of Internal Medicine, Occupational Diseases and Hypertension, Wroclaw Medical University, 50-556 Wroclaw, Poland; helena.martynowicz@umw.edu.pl (H.M.); grzegorz.mazur@umw.edu.pl (G.M.); rafal.poreba@umw.edu.pl (R.P.)

**Keywords:** arterial hypertension, left ventricle hypertrophy, serum selenium concentrations, serum zinc concentration

## Abstract

The aim of the study was to assess the relationship between serum selenium and zinc concentrations (Se-S and Zn-S) and the left ventricle geometry in patients suffering from arterial hypertension. A total of 78 people with arterial hypertension (mean age: 53.72 ± 12.74 years) participated in the study. Se-S and Zn-S were determined in all patients. The type of left ventricular remodelling and hypertrophy was determined by the left ventricular mass index (LVMI) and relative wall thickness (RWT) measured by echocardiography. Se-S and Zn-S in the whole group were 89.84 ± 18.75 µg/L and 0.86 ± 0.13 mg/L. Normal left ventricular geometry was found in 28.2% of patients; left ventricular hypertrophy (LVH) in 71.8%, including concentric remodelling in 28.2%, concentric hypertrophy in 29.5%, and eccentric hypertrophy in 14.1%. LVH was statistically significantly more frequent in patients with Se-S < median compared to patients with Se-S ≥ median (87.2% vs. 56.4%, *p* < 0.05), as well as in patients with Zn-S < median compared to patients with Zn-S ≥ median (83.8% vs. 60.9%, *p* < 0.05). In hypertensive patients, older age, higher LDL cholesterol, higher fasting glucose, lower Se-S, and lower Zn-S were independently associated with LVH. In conclusion, in hypertensive patients, left ventricular hypertrophy may be associated with low levels of selenium and zinc in the serum.

## 1. Introduction

Selenium and zinc are essential micronutrients that have a huge impact on human health. They are incorporated in many enzymes and play a key role in optimising metabolic reactions and protecting against oxidative damage [1,2,3,4]. It is estimated that humans have about 25 different Se-dependent enzymes (selenoproteins); the best known include glutathione peroxidase (GPx), iodothyronine deiodinases (IDD) and thioredoxin reductase (TrxR). Multiple studies suggest that they are essential for maintaining successful reproduction and proper function of the brain, thyroid and immune system [1,2]. Zinc is the core of human metabolic processes; it acts as a cofactor for over 300 enzymes and 2000 transcription factors. In addition to its’ antioxidant properties, it also regulates cell physiology, gene transcription, insulin secretion and serves as an extracellular signalling molecule [3,4].

Scientific reports analysing the impact of selenium and zinc blood concentrations on arterial hypertension (HT) are inconsistent [5]. The only confirmed correlation concerns low blood selenium concentration and HT in Keshan disease. Symptoms of the disease, which include cardiac dysfunction, hypertension and arrhythmias, were proven to withdraw with selenium supplementation [6]. The other reports are conflicting, some show no association between HT and blood selenium or zinc concentrations [7,8], whereas some indicate that such correlation exists. What is more, there is no agreement on the correlation type, several studies discovered higher blood selenium and zinc concentrations in HT patients [9,10,11], whereas others found contrary results [12,13].

Hypertension highly contributes to multiple changes in left ventricle physiology and geometry [14,15,16,17,18,19]. Chronic pressure overload challenges the heart muscle and triggers various molecular and cellular processes that lead to ventricular remodelling [14,15,16]. These processes include interstitial fibrosis and inflammation, cardiomyocyte hypertrophy and death, endothelial dysfunction with vascular stiffness and consequently ventricular wall-thickening [14,15]. These changes seem to be a natural adaptation of the heart to the increased wall tension, because (according to Laplace’s law) the tension of the ventricular wall is inversely proportional to the wall thickness [14,16]. However, persistent pressure overload can lead to irreversible organ damage, decompensation and clinical heart failure [17]. It should be noted that ventricle remodelling results in different left ventricle geometry; namely, three different geometric patterns are described: concentric remodelling (CR), concentric hypertrophy (CH) and eccentric hypertrophy (EH). Assessment of the geometric changes can be performed using electrocardiography, echocardiography and cardiac magnetic resonance [18]. Interestingly, many hypertensive patients do not present clinically detectable changes in LV geometry. In this regard, scientists suggest that hypertrophy results from pressure overload and some other factors, such as sex, age, environmental exposures, chronic diseases and genetic factors [15,19].

The amount of evidence for the role of oxidative stress in the pathophysiology of both hypertension and heart remodelling or hypertrophy is growing [20,21,22,23]. In general, optimal intracellular concentrations of reactive oxygen species are essential to regulate various signalling pathways and maintain vascular function [24]. However, overproduction of ROS and their incomplete neutralisation cause oxidative damage to cellular elements. Many factors seem to intensify cardiac mitochondrial function and thus increase ROS production in hypertension; these include general sympathetic stimulation, increase in heart rate per second and overstimulation of the renin-angiotensin system [25]. When ROS production overtakes cellular defence mechanisms a broad variety of signalling proteins get activated, namely non-receptor proteins kinases (PTKs), protein phosphatases (PTPs), Mitogen-activated Protein Kinases (MAPKS) and Nuclear Factor ƙB (NF- ƙB) [22]. They all play a significant role in cardiac remodelling evolution. However, there are enzymes such as superoxide dismutase (SOD) and its isoenzymes (CuZnSOD and MnSOD), catalase, glutathione reductase and peroxidase that protect myocytes from oxidative damage [26,27]. There are two main selenoproteins involved in fighting cardiac stress: plasma glutathione peroxidase (GPX3) and phospholipid hydroperoxide glutathione peroxidase (GPX4) [26]. Both were found to be upregulated during myocardial hypertrophy induced with either thyroid hormone or isoproterenol treatment [28]. In addition, the expression of thioredoxin-1 (Se-containing enzyme involved in redox signalling pathways) was also found to be increased during hypertrophy [28]. However, their exact mechanism of action in cardiac hypertrophy and their correlation with blood Se are yet to be determined [26,29,30].

The aim of the study was to assess the relationship between serum selenium and zinc concentrations (Se-S and Zn-S) and the left ventricle geometry in patients suffering from arterial hypertension.

## 2. Materials and Methods

The present study was part of a research project entitled Selenium and zinc deficiencies as predictors of subclinical cardiovascular complications in hypertension (STM.A100.17.050). The project was approved by the institutional bioethics committee. The respondents gave their informed written consent to participate in the study.

Originally, the study comprised 141 patients with arterial hypertension, meeting the assumed criteria of inclusion in the study: age ≥ 18 years, diagnosed and pharmacologically treated arterial hypertension and no modification in the treatment of arterial hypertension in the last year prior to study enrolment. The next stage was to exclude patients, whose medical history included ischaemic heart disease, stroke, type-2 diabetes, peripheral artery disease and chronic renal disease. The 78 patients remaining after this stage of qualification for the study formed the proper study group. The clinical characteristic of the study group is presented in Table 1.

The study methodology encompassed anamnesis and physical examination, laboratory tests and echocardiography. The anamnesis and physical examination of all the study patients provided personal data, information on any drugs and stimulants use, health conditions, as well as information on the arterial hypertension treatment. The lipid profile and the fasting serum glucose concentration were determined by standard commercially available tests. The serum selenium concentration (Se-S) was determined using atomic absorption spectroscopy in combination with the hydride generation atomic absorption spectroscopy (HGAAS) technology. The serum zinc concentration (Zn-S) was determined with the flame atomic absorption spectroscopy (FAAS) technology. The laboratory for determining Se-S and Zn-S has a certificate of proficiency in the determination of selenium in blood issued by the CDC in Atlanta (Lead and Multielement Proficiency LAMP Program) and a certificate of proficiency in the determination of zinc in serum obtained under the German External Quality Assessment Scheme (G_EQUAS). Se-S and Zn-S were determined using the typical methodology, described previously [31]. The study patient group was divided by cut-off points constituting the medians of the serum selenium concentration and the serum zinc concentration. Based on the Se-S median (88.91 μg/L), a subgroup of patients with low Se (Se-S less than the median) and a subgroup of patients with high Se-S (Se-S equal to or greater than the median) we created. Based on the Zn-S median (0.84 mg/L), a subgroup of patients with low Zn (Zn-S less than the median) and a subgroup of patients with high Zn-S (Zn-S equal to or greater than the median) were created.

The echocardiography scan was performed using the transthoracic approach, with ALOKA ProSound 6 (Aloka Inc., Tokyo, Japan), 3.5/2.7 MHz head. In the M-mode presentation acquired under control of a 2D scan (parasternal view, left ventricle long axis), the left ventricular end-diastolic diameter (LVEDd) and the left ventricular end-systolic diameter (LVESd) were measured, along with the intraventricular septum diastolic diameter (IVSDd) and the posterior wall diastolic diameter (PWDd). Ejection fraction (EF) was determined with biplane Simpson’s method. The left ventricular mass (LVM) was estimated using the mathematical formula: LVM = 0.8 × [1.04 × (LVEDd + PWDd + IVSDd)^3^ − LVEDd^3^] + 0.6. The left ventricle mass index (LVMI) was calculated by dividing the LVM value by the body surface area (BSA). When calculating BSA, the Dubois formula was used: BSA = 0.007184 × body mass ^0.425^ × height ^0.725^. The relative wall thickness (RWT) was obtained by applying the formula: RWT = (IVSDd + PWDd)/LVEDd.

Based on the LVMI and RWT values acquired from the echocardiography, the study group was divided into patients with normal left ventricle geometry (NG): RWT ≤ 0.45 and LVMI ≤ 125 g/m^2^ in males and ≤110 g/m^2^ in females; patients with left ventricle hypertrophy (LVH): RWT > 0.45 and/or LVMI > 125 g/m^2^ in males and >110 g/m^2^ in females. The resulting LVMI and RWT values were also used to isolate the types of the left ventricle remodelling and hypertrophy: concentric remodelling (CR): RWT > 0.45 and LVMI ≤ 125 g/m^2^ in males and ≤110 g/m^2^ in females; concentric hypertrophy (CH): RWT > 0.45 and LVMI ≤ 125 g/m^2^ in males and ≤110 g/m^2^ in females; and eccentric hypertrophy (EH): RWT ≤ 0.45 and LVMI > 125 g/m^2^ in males and >110 g/m^2^ in females.

Statistical analysis was performed using the statistical software “Dell Statistica 13” (Dell Inc., Round Rock, TX, USA). The distribution of variables was verified using the Lillefors and W Shapiro–Wilk tests. In the case of quantitative independent variables with normal distribution, further statistical analysis was performed using the *t*-test for independent variables or ANOVA (univariate, parametric). For variables with an abnormal distribution for independent quantitative variables, the Mann–Whitney U test or the Kruskal–Wallis ANOVA, was used. Statistically significant differences between means were determined using the Newman–Keuls post-hoc test. The results for quantitative variables are expressed as a percentage. For independent qualitative variables, further statistical analysis was performed using the Chi-square test. To determine the relationship between the studied variables, correlation and regression analysis was performed. Moreover, the validity of the test was assessed based on the analysis of ROC (Receiver Operating Characteristic) curves. The results *p* < 0.05 were considered statistically significant.

## 3. Results

In the study group, the Se-S and Zn-S results were 89.84 ± 18.75 µg/L and 0.86 ± 0.13 mg/L, respectively. The Se-S values were observed within the range of 53.76 µg/L to 182.05 µg/L, with a median of 88.91 µg/L. The range of the observed Zn-S values was 0.52 mg/L to 1.21 mg/L, with the Zn-S median of 0.84 mg/L. The results of the laboratory determinations in the study group are presented in Table 2.

The study group was characterised by a normal systolic function of the left ventricle in the echocardiography examination. The ejection fraction was 67.13 ± 5.13%, staying within the range of 58–77% in individual patients. In the study group, the LVMI was 114.83 ± 36.44 g/m2, and the RWT was —0.46 ± 0.06. Normal left ventricle geometry was observed only in 28.2% of the subjects, with the left ventricle hypertrophy in 71.8%. Among the patients with left ventricle hypertrophy, concentric remodelling occurred in 28.2%, concentric hypertrophy in 29.5%, and eccentric hypertrophy in 14.1%. The results of the echocardiograph tests for the study group are presented in Table 3.

When comparing the patient subgroups divided by the median of the serum selenium concentration and the median of the serum zinc concentration, it was demonstrated that LVH was statistically significantly more frequent in patients with Se-S < median compared to patients with Se-S ≥ median (87.2% vs. 56.4%, *p* < 0.05), as well as in patients with Zn-S < median compared to patients with Zn-S ≥ median (83.8% vs. 60.9%, *p* < 0.05). Geometry and hypertrophy of the left ventricle in the subgroups with different serum selenium and zinc concentrations are presented in Table 4.

When comparing the subgroups divided by the type of geometry and hypertrophy of the left ventricle, it was found that in patients with LVH there are statistically significantly lower Se-S and Zn-S values than in patients with normal left ventricle geometry (Se-S—85.76 ± 13.19 µg/L vs. 100.24 ± 26.01 µg/L, Zn-S—0.83 ± 0.13 mg/L vs. 0.90 ± 0.12 mg/L, *p* < 0.05). Moreover, the Se-S was statistically significantly lower in the patient subgroups with concentric remodelling and concentric hypertrophy, compared to the subgroup with a normal left ventricle geometry (CR: 84.10 ± 12.89 µg/L, CH: 85.11 ± 12.41 µg/L, NG: 100.24 ± 26.01 µg/L, *p* < 0.05). Whereas the Zn-S was statistically significantly lower in the subgroup with eccentric hypertrophy, compared to the subgroup with a normal left ventricle geometry ((0.80 ± 0.14 mg/L vs. 0.90 ± 0.12 mg/L, *p* < 0.05). Se-S and Zn-S in subgroups with different types of the left ventricle geometry and hypertrophy are presented in Table 4.

**Figure 1:**
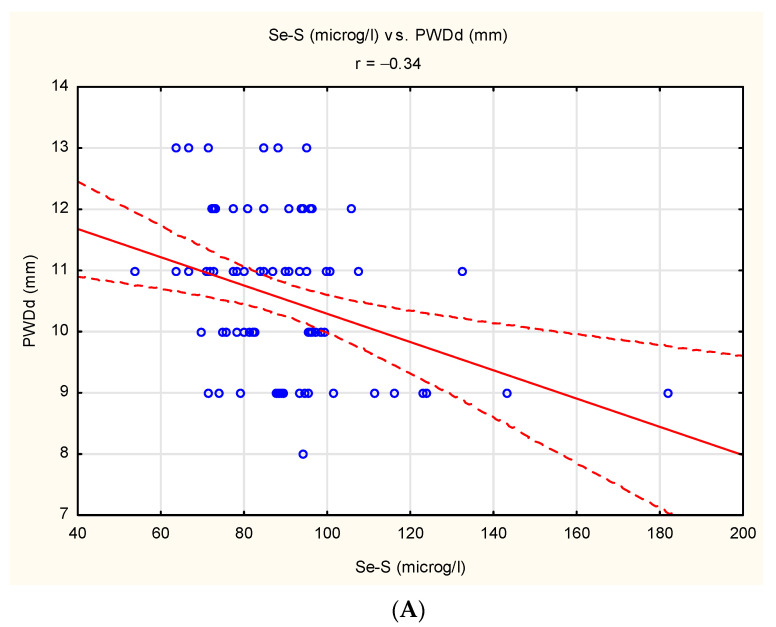

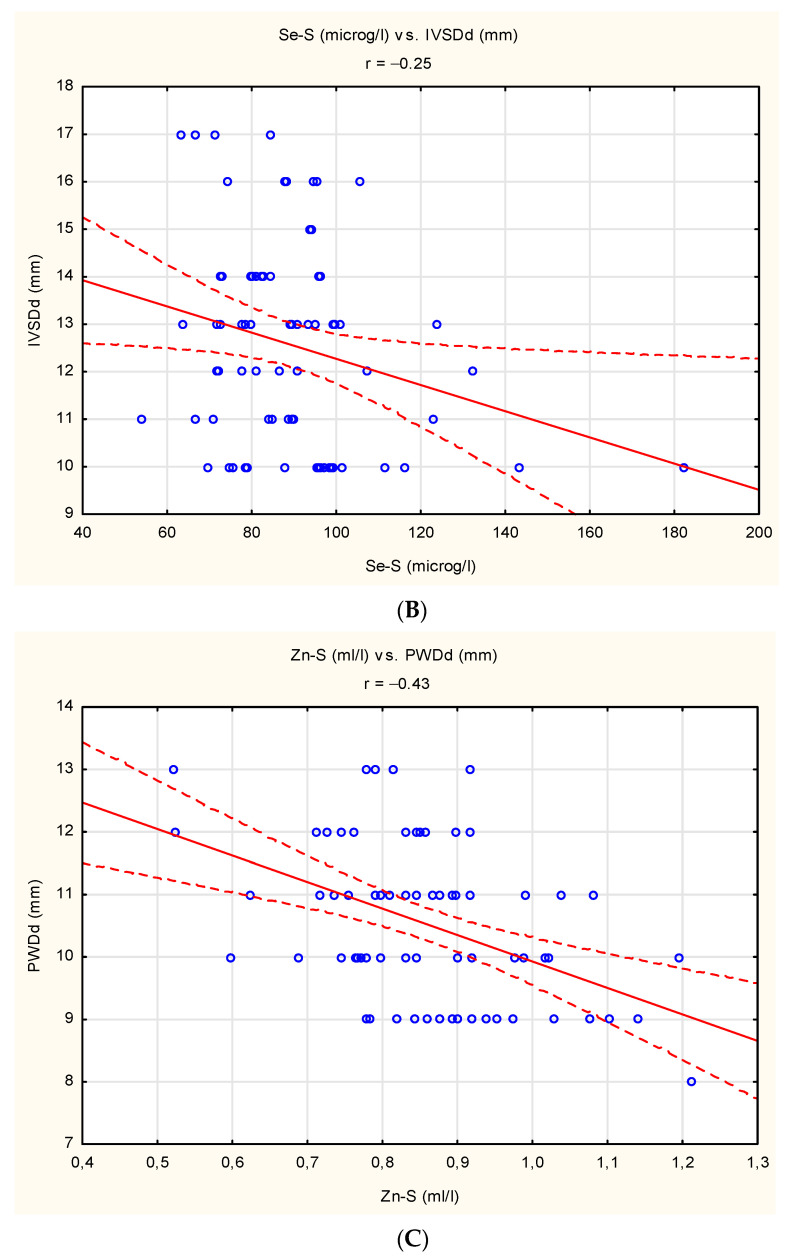
Statistically significant correlations in study group: (**A**) between Se-S and PWDd (r = −0.34, *p* < 0.05). (**B**) between Se-S and IVSDd (r = −0.25, *p* < 0.05). (**C**) between Zn-S and PWDd (r = −0.43, *p* < 0.05).

The correlation analysis demonstrated the existence of negative linear relationship between Se-S and PWDd (r = −0.34, *p* < 0.05), between Se-S and IVSDd (r = −0.25, *p* < 0.05) and between Zn-S and PWDd (r = −0.43, *p* < 0.05). The documented correlations are presented in Figure 1A–C.

An analysis of the logistic regression performed using the hierarchical method, considering the basic anthropological parameters (age, gender, BMI), the laboratory parameters (lipids, glucose, Se-S and Zn-S) and smoking, resulted in the following model: logit LVH = −0.92 + 0.07 age + 0.04 LDL cholesterol + 0.03 glucose—0.03 Se-S—0.22 Zn-S.

Based on this model, it was demonstrated that independent risk factors of LVH in our study group are older age, higher LDL cholesterol, higher fasting glucose, lower Se-S and lower Zn-S (OR age = 1.07, OR LDL cholesterol = 1.08, OR glucose = 1.07, OR Se-S = 0.96, OR Zn-S = 0.92, *p* < 0.05). The parameters of the model acquired from the regression analysis are presented in Table 5.

**Figure 2:**
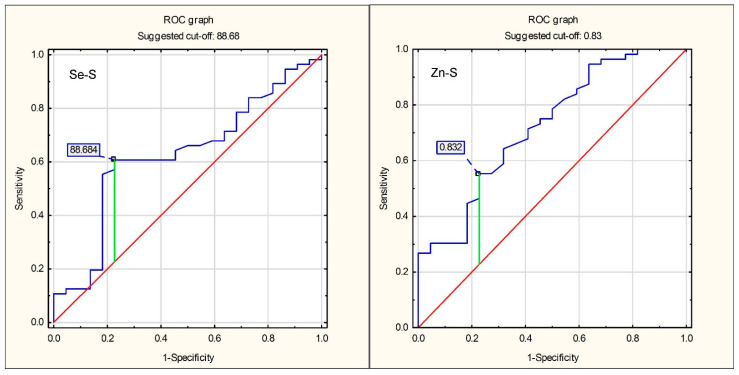
ROC curves for prediction of left ventricular hypertrophy using serum selenium concentration and serum zinc concentration. The blue line is the ROC curve of the assessed variables, the red line is the diagonal reference line.

To determine the optimum cut-off points for the LVH prediction, ROC curves were plotted. For Se-S, the optimum cut-off point for the LVH prediction was 88.68 µg/L, whereas for Zn-S this cut-off point was 0.83 mg/L. The ROC curves are presented in Figure 2.

The sensitivity, specificity and accuracy of the criterion Se-S < 88.86 µg/L as a LVH predictor were 77.3%, 58.9% and 64.1%, respectively; the analogous parameters of the accuracy analysis for the criterion Zn-S < 0.83 mg/L as an LVH predictor were 77.3%, 55.4% and 61.5%, respectively. The complete parameters of accuracy analysis for Se-S and Zn-S as the left ventricle hypertrophy predictors are shown in Table 6.

## 4. Discussion

In our study, we showed that in hypertensive patients, left ventricular hypertrophy may be associated with a low level of selenium in the serum. Our observation is important in the aspect of previous research on the relationship between selenium concentration in blood and echocardiographic changes. Up to date, the relationship between echocardiographic changes and blood selenium concentration remains unknown; moreover, the data on the subject are limited and conflicting. The most attention was paid to the relationship between selenium concentration and the systolic function of the left ventricle, assessed based on the ejection fraction (LVEF). A recent case-control study by Miramadi et al. [32] showed no significant correlation between blood Se and LVEF in patients suffering from heart failure. Similar results were found by other scientific groups, inter alia, Frączek-Jucha et al. (2019) [33], Ghaemian et al. (2012) [34] and De Lorgeril et al. (2001) [35]. Likewise, studies on patients suffering from idiopathic dilated cardiomyopathy yielded no such correlation [36]. On the contrary, some scientists reported a positive linear relationship between blood selenium level and LVEF [37,38]. A significant change in cardiac function was also reported among Swedish citizens aged 70 to 88 years who were given combined selenium and coenzyme Q10 supplementation. A total of 228 participants who completed the study took 200 mg/day of coenzyme Q10 capsules and 200 μg/day of organic selenium yeast tablets or matching placebo tablets for 48 months. At the end of the study reduced cardiovascular mortality and improvement in cardiac function were reported [39]. However, these results stand against previous research which does not support the notion that Se supplementation decreases cardiovascular mortality [30].

Scientific data concerning echocardiographic parameters other than LVEF are very limited. Mirdamadi et al. found a significant inverse relationship between the concentration of selenium and the volume of the left ventricle and pressure in the pulmonary artery in patients with heart failure (HF). As they concluded this may show the effect of selenium deficiency on HF; when selenium level decreases, oxidative stress will increase and high blood pressure increases the volume of the left ventricle [32]. Nevertheless, the sample size of this research was rather small with only 32 patients in the study group. This is important while interpreting the result for pulmonary artery pressure because recent studies suggest that the development and progression of pulmonary artery hypertension (PAH) are mostly related to upregulation of Selenoprotein P (SeP) in the distal pulmonary arteries and not with blood Se level [40,41]. SeP is a secreted glycoprotein that contains multiple selenocysteine (SeC) residues. It is thought to possess two functions: enzyme activity (antioxidation) and selenium-supply activity (intercellular transport or storage) [42]. Interestingly, a mutated SeP with no selenium content was found to be overexpressed in PAH patients. The enhanced expression of SeP increased oxidative stress levels, contributing to the enhanced proliferation of pulmonary artery smooth muscle cells (PASMCs) and these changes had no relation to selenium content in SeP [40]. It should be clearly emphasised that our study concerned morphological changes of the heart, in the studies published so far, functional changes were assessed. To dispel all doubts and conflicting results regarding the effect of blood selenium on cardiac morphology and function more advanced studies with a broader spectrum of echocardiographic and laboratory parameters need to be carried out.

In addition, the present study documented that in hypertensive patients, left ventricular hypertrophy may be associated with a low level of zinc in the serum. Our study provides important data, so far studies on the relationship between zinc concentration and echocardiographic changes were even less numerous than in the case of selenium studies. However, as with selenium, they were inconclusive. Echocardiographic correlates of serum zinc concentration were recently assessed by Yoshihisa et al. in 968 consecutive hospitalised patients with decompensated heart failure [43]. The investigated echocardiographic parameters included left ventricular ejection fraction, change in right ventricular fraction area, pressure gradient for tricuspid regurgitation and inferior vena cava diameter. None of these parameters was found to be significantly correlated with Zn-S. Moreover, these results were consistent with a previous study by Ioannis et al. who also claimed no significant correlation between LVEF with Zn-S in heart failure patients [44]. However, both studies described significantly lower Zn-S in heart failure patients when compared to healthy controls [43,44]. Whereas Huang et al. found that zinc concentration differs among patients with different left ventricle geometry patterns [45]. In this study, zinc concentrations were decreased in the LVH patients when compared with the control group (641.9 ± 215.2 μg/L and 710.2 ± 243.0 μg/L). Further analyses showed that zinc concentration in eccentric hypertrophy and concentric hypertrophy groups was significantly decreased compared to normal left ventricle geometry. Furthermore, regression analyses were performed to confirm the relationship between Zn concentration and left ventricle geometry. There was a significant inverse linear relationship between Zn and left ventricle mass and left ventricle mass index but no significant relationship with relative wall thickness [45]. Our results are consistent with those obtained by Huang et al., but in the context of the results of other researchers, further research in this aspect will also be justified.

Finally, based on our research, it can be concluded that lower serum selenium and zinc concentrations may be predictors for left ventricle hypertrophy in patients with arterial hypertension. Knowing the serum selenium and zinc concentration, it is possible to predict with a certain probability the risk of left ventricular hypertrophy in people suffering from arterial hypertension. In the studies conducted so far, a similar hypothesis has not been verified.

The lack of a control group of people without hypertension is a main limitation of the study. The optimal methodological approach would be to include in the study group, in addition to patients with arterial hypertension, also healthy people. The selection of our group was limited by the place of recruitment of patients. The research material was collected in the hypertensiology clinic during hospitalisation of patients with arterial hypertension to verify the effectiveness of the disease therapy and to optimise this therapy. Moreover, the aim of our study was not to compare patients with arterial hypertension with patients without hypertension. Research hypotheses included the comparison of patients with left ventricular hypertrophy with patients without left ventricular hypertrophy, and patients with lower serum selenium concentrations with patients with higher serum selenium concentrations, and patients with lower serum zinc concentrations with patients with higher serum zinc concentrations. Hence, the control groups in the present study (at such ranges of serum selenium and zinc concentrations as were shown in the study group) in subsequent comparisons were patients with normal left ventricular geometry, patients with higher serum selenium concentrations, as well as patients with higher serum selenium concentrations. serum zinc concentrations. A significant methodological limitation of the study is the lack of laboratory determinations of matrix metalloproteases. Their determination could indicate an important pathomechanism explaining the relationships that were observed in our study. The lack of these determinations resulted from the limited volume of the obtained biological material.

To sum up, it should be noted that in the light of our research results (showing a significant relationship between serum selenium and zinc concentrations and left ventricular hypertrophy in patients with arterial hypertension), greater attention should be paid to the therapeutic importance of a healthy lifestyle and nutrition.

## 5. Conclusions

Patients with arterial hypertension and left ventricle hypertrophy demonstrate lower serum selenium and zinc concentrations compared to patients with arterial hypertension and normal left ventricle geometry.

Lower Se-S and Zn-S, regardless of older age, higher LDL cholesterol concentration and higher glucose concentration, are independent risk factors of left ventricular hypertrophy in patients with arterial hypertension.

Lower serum selenium and zinc concentrations may be predictors for left ventricle hypertrophy in patients with arterial hypertension.

In patients with arterial hypertension, more attention should be paid to non-pharmacological methods of therapy, i.e., optimisation of lifestyle and diet.

## Figures and Tables

**Table 1:** Clinical characteristics of the study group.

age (years) ^#^	53.72 ± 12.74
height (cm) ^#^	172.72 ± 10.88
body mass (kg) ^#^	95.17 ± 18.03
BMI (kg/m^2^) ^#^	31.79 ± 4.67
smoking *	20.5 (16)
pack-years of smoking ^#^	21.15 ± 8.15
arterial hypertension *	100.0 (78)
duration of arterial hypertension (years) ^#^	11.86 ± 9.32
diuretics *	51.3 (40)
β-blockers *	51.3 (40)
ACE inhibitors *	35.9 (28)
angiotensyn receptor blockers *	42.3 (33)
calcium channel blockers *	51.3 (40)

^#^ mean ± standard deviation; * percentage (number); ACE—angiotensin-converting enzyme; BMI—body mass index.

**Table 2:** Laboratory tests in the study group.

total cholesterol (mg/dl) ^#^	204.80 ± 44.40
HDL cholesterol (mg/dl) ^#^	48.83 ± 13.33
LDL cholesterol (mg/dl) ^#^	121.00 ± 35.88
triglicerides (mg/dl) ^#^	197.66 ± 144.51
glucose (mg/dl) ^#^	100.12 ± 21.88
Se-S (μg/L) ^#^	89.84 ± 18.75
Zn-S (mg/L) ^#^	0.86 ± 0.13

^#^ mean ± standard deviation; HDL—high-density lipoprotein; LDL—low-density lipoprotein; Se-S—serum selenium concentration; Zn-S—serum zinc concentration.

**Table 3:** Echocardiographic parameters in the study group.

LVEDd (mm) ^#^	50.67 ± 5.56
LVESd (mm) ^#^	30.85 ± 3.83
IVSDd (mm) ^#^	12.55 ± 2.11
PWDd (mm) ^#^	10.54 ± 1.26
EF (%) ^#^	67.13 ± 5.13
LVMI (g/m^2^) ^#^	114.83 ± 36.44
RWT ^#^	0.46 ± 0.06
NG *	28.2 (22)
LVH *	71.8 (56)
CR *	28.2 (22)
CH *	29.5 (23)
EH *	14.1 (11)

^#^ mean ± standard deviation; * percentage (number); CH—concentric hypertrophy of the left ventricle; CR—concentric remodelling of the left ventricle; EF—ejection fraction of the left ventricle; EH—eccentric hypertrophy of the left ventricle; IVSDd—interventricular septum diastolic diameter; LVEDd—left ventricle end-diastolic diameter; LVESd—left ventricle end-systolic diameter; LVH—left ventricle hypertrophy; LVMI—left ventricle mass index; NG—normal geometry of the left ventricle; PWDd—posterior wall diastolic diameter; RWT—relative wall thickness.

**Table 4:** Geometry and hypertrophy of the left ventricle and serum selenium and zinc concentrations in the studied subgroups. (**A**) Geometry and hypertrophy of the left ventricle in the studied subgroups differ in serum selenium and zinc concentrations. (**B**) Serum selenium and zinc concentrations in the studied subgroups differ in geometry and left ventricular hypertrophy.

A	Subgroups Differing in Se-S			Subgroups Differing in Zn-S		
	Low-Se (Se-S < 88.91 μg/L)	High-Se (Se-S ≥ 88.91 μg/L)	*p*	Low-Zn (Zn-S < 0.84 mg/L)	High-Zn (Zn-S ≥ 0.84 mg/L)	*p*
NG *	12.8 (5)	43.6 (17)	0.003 &	16.2 (6)	39.0 (16)	0.025 &
LVH *	87.2 (34)	56.4 (22)	0.003 &	83.8 (31)	60.9 (25)	0.025 &
CR *	30.8 (12)	25.6 (10)	0.609	32.4 (12)	24.4 (10)	0.431
CH *	35.9 (14)	23.1 (9)	0.215	32.4 (12)	26.8 (11)	0.587
EH *	20.5 (8)	7.7 (3)	0.104	18.9 (7)	9.8 (4)	0.248
**B**			**Se-S (μg/L) ^#^**		**Zn-S (mg/L) ^#^**	
subgroups differing in left ventricular hypertrophy		NG	100.24 ± 26.01		0.90 ± 0.12	
		LVH	85.76 ± 13.19		0.83 ± 0.13	
		*p*	0.002 &		0.032 &	
subgroups differing in the type of left ventricular geometry		NG	100.24 ± 26.01		0.90 ± 0.12	
		CR	84.10 ± 12.89		0.83 ± 0.14	
		CH	85.11 ± 12.41		0.85 ± 0.12	
		EH	90.44 ± 15.44		0.80 ± 0.14	
		*p*	NG vs. CR: 0.004 &		NG vs. CR: 0.174	
			NG vs. CH: 0.006 &		NG vs. CH: 0.376	
			NG vs. EH: 0.140		NG vs. EH: 0.037 &	
			CR vs. CH: 0.849		CR vs. CH: 0.916	
			CR vs. EH: 0.338		CR vs. EH: 0.521	
			CH vs. EH: 0.416		CH vs. EH: 0.428	

^#^ mean ± standard deviation; * percentage (number); & statistically significant difference (*p* < 0.05); CH—concentric hypertrophy of the left ventricle; CR—concentric remodelling of the left ventricle; EH—eccentric hypertrophy of the left ventricle; LVH—left ventricle hypertrophy; NG—normal geometry of the left ventricle; Se-S—serum selenium concentration; Zn-S—serum zinc concentration.

**Table 5:** Results of logistic regression analysis in the study group.

	Model for: Probability of Left Ventricle Hypertrophy					
	Intercept	Age (Years)	LDL Cholesterol (mg/dl)	Glucose (mg/dl)	Se-S (μg/L)	Zn-S (mg/L)
regression coefficient	−0.92	0.07	0.04	0.03	−0.03	−0.22
SEM of Rc	0.40	0.02	0.02	0.01	0.01	0.10
*p* value	<0.01 &	0.04 &	0.03 &	0.04 &	0.02 &	0.01 &
odds ratio (for unit change)	0.09	1.07	1.08	1.07	0.96	0.92
confidence interval						
−95%	0.03	1.01	1.01	1.02	0.91	0.89
+95%	0.11	1.15	1.16	1.11	0.98	0.96

& statistically significant (*p* < 0.05); LDL—low density lipoprotein; Rc—regression coefficient; SEM—standard error of mean; Se-S—serum selenium concentration; Zn-S—serum zinc concentration.

**Table 6:** Sensitivity and specificity of serum selenium concentration and serum zinc concentration as predictors of left ventricular hypertrophy.

	LVH Prediction	
	Se-S < 88.68 μg/L	Zn-S < 0.83 mg/L
sensitivity	0.773	0.773
specificity	0.589	0.554
accuracy	0.641	0.615
positive prediction value	0.425	0.405
negative predictive value	0.868	0.861
positive likelihood ratio	1.881	1.731
negative likelihood ratio	0.386	0.411

LVH—left ventricle hypertrophy; Se-S—serum selenium concentration; Zn-S—serum zinc concentration.

## Data Availability

The data presented in this study are available in this manuscript.
